# Serum Levels of ApoA1 and ApoA2 Are Associated with Cognitive Status in Older Men

**DOI:** 10.1155/2015/481621

**Published:** 2015-11-23

**Authors:** Cheng Ma, Jin Li, Zhijun Bao, Qingwei Ruan, Zhuowei Yu

**Affiliations:** ^1^Department of Geriatrics, Huadong Hospital, Shanghai Medical College, Fudan University, Shanghai 200040, China; ^2^Department of Aging, Antiaging and Cognitive Function, Shanghai Institute of Geriatrics and Gerontology, Shanghai 200040, China; ^3^Research Center of Aging and Medicine, Department of Aging and Shanghai Key Laboratory of Clinical Geriatrics, Huadong Hospital, Shanghai Medical College, Fudan University, Shanghai 200040, China

## Abstract

*Background.* Advancing age, chronic inflammation, oxidative damage, and disorders of lipid metabolism are positively linked to the late-life cognitive impairment. Serum biomarkers may be associated with the cognitive status in older men.* Methods.* 440 old male subjects with different cognitive functions were recruited to investigate probable serum markers. Pearson Chi-Squared test, univariate analysis, and multivariate logistic regression analysis were performed to evaluate biomarkers which may be associated with cognitive status.* Results.* Levels of fundus atherosclerosis (AS) (*P* < 0.001), age (*P* < 0.001), serum biomarkers peroxidase (POD) (*P* = 0.026) and interleukin-6 (IL-6) (*P* = 0.001), serum levels of high-density lipoprotein cholesterol (HDL-C) (*P* < 0.001), apolipoprotein A2 (ApoA2) (*P* = 0.001), and ApoC2 (*P* = 0.005) showed significant differences. Compared to group 3, ApoA1 in group 1 (OR = 1.30, 95% CI 1.01–1.67) and group 2 (OR = 1.47, 95% CI 1.11–1.94) were higher, while ApoA2 were lower (group 1: OR = 0.43, 95% CI 0.18–1.02; group 2: OR = 0.21, 95% CI 0.08–0.54) after adjusting for control variables.* Conclusion.* The results demonstrated that age, AS levels, POD, IL-6, HDL-C, ApoA2, and ApoC2 were significantly related to cognitive status. Moreover, ApoA1 and ApoA2 were independently associated with cognitive impairment and late-life dementia.

## 1. Introduction

The decline of cognitive function across the life span is referred to as age-related cognitive decline [1, 2]. In addition to age, many age-related health problems, such as sex, general medical health, heart disease, vascular diseases, hypertension, stroke, diabetes, high plasma cholesterol, and a high number comorbidities, are recognized as risk factors of cognitive decline [3–7]. Other susceptibility factors, including antioxidant status, inflammatory status, and apolipoproteins, result in a substantial variation in the rate of cognitive decline among older individuals in the population [8–10]. The pathology of neurodegeneration in cognitive function impairment, Alzheimer's disease (AD), and vascular dementia (VaD) involves oxidative stress and the accumulation of free radicals [8, 11]. The antioxidants selective monoamine oxidase inhibitor selegiline and alpha-tocopherol (vitamin E) can slow cognitive decline in patients with moderately severe impairment from AD [11]. The polymorphism of antioxidant genes had been demonstrated to be a risk factor for late-onset AD [12, 13]. The antioxidant status can predict neurodegeneration, such as medial temporal lobe atrophy in AD. A small drop in the antioxidant concentration increases the risk of medial temporal lobe atrophy in AD [8]. However, some clinical trials with antioxidants had largely been disappointing. The long-term use of* Ginkgo biloba* extract did not reduce the risk of progression to AD [14]. The use of vitamin E could maintain the cognitive status in some AD individuals and, meanwhile, accelerate the cognitive decline in other AD individuals [15]. Both chronic low-grade inflammation and immune activation are playing important roles in the pathogenesis of age-associated diseases, including cognitive decline, AD, and vascular dementia. Serum or plasma biomarkers of inflammation, especially IL-6 and C-reactive protein (CRP), were prospectively associated with cognitive decline in multiethnic cohorts of older populations before the clinical onset of dementia, AD, and vascular dementia [16–20]. The level of IL-6 was negatively associated with the performance on the Minimental Status Examination (MMSE) in a stroke-free population-based multiethnic cohort, including Hispanic and black subjects; after adjusting for vascular disease and subclinical atherosclerosis (AS), the association did not attenuate [21]. The IL-6-174 G/C polymorphism (C allele caused increase of IL-6) was associated with worse cognitive function and steeper cognitive decline in participants in the Prospective Study of Pravastatin in the Elderly at Risk (PROSPER) [22]. Increased plasma IL-6 level also represented a biomarker for the risk of future cognitive decline in a relatively healthy midlife (age from 30 to 54) community sample [23]. In another large-scale, prospective occupational cohort study over 10 years, increased levels of inflammatory markers, both CRP and IL-6 at midlife, were moderately associated with a lower cognitive status but were only slightly associated with cognitive decline [24]. Systemic inflammation was associated with mild cognitive impairment (MCI) and its subtypes. The level of plasma TNF-*α* was higher in participants with MCI compared to individuals with normal cognitive function [25]. Nonamnestic multiple domains MCI was associated with higher levels of IL-1*β*, IL-12, and TNF-*α* compared with normal cognition, amnestic MCI (single and multiple domains), and nonamnestic single-domain MCI [25]. However, the systemic inflammation markers CRP and IL-6 were not associated with dynamic cognitive decline in amnestic MCI after a follow-up over 12 months [26].

Accumulating evidence suggests that serum lipid levels are associated with human longevity, cognitive decline, and dementia [27, 28]. Serum high-density lipoprotein cholesterol (HDL-C) level was associated with better survival in the frail community-living elderly [29]. Higher level of HDL-C indicated better functional status in individuals who were over 85 years old [30] and less use of prescribed drugs also appeared in nonagenarians [31]. Low-density lipoprotein cholesterol (LDL-C) level was related to male longevity, while triglycerides (TG) level was related to female in offspring of nonagenarian siblings (*n* = 1,664, mean age: 59 years) when compared to offspring of control population (*n* = 711, mean age: 60 years) [32]. In a longitudinal study (*n* = 1,449) with average follow-up of 21 years, the results showed that the association between serum total cholesterol (TC) levels and dementia might be bidirectional. High midlife serum TC represented a risk factor for subsequent dementia, and low serum TC after midlife was a risk factor for late-life cognitive impairment [33]. Low serum cholesterol and LDL-C levels were closely related to cognitive decline in elderly patients with Alzheimer's disease (AD) [28]. Subjects with probable mild cognitive impairment (MCI) and AD had significantly lower levels of TG, TC, or LDL-C in elderly people [34, 35].

Apolipoproteins are important components of plasma lipoproteins which are synthesized in liver and have been proved to play a significant role in the lipid metabolism and the formation of lipoproteins [36]. Some recent studies showed that apolipoproteins are potential plasma biomarkers of cognitive decline and then AD in older adults [37]. ApoA1 and ApoA2, in charge of transporting cholesterol to the liver, are critical components in the formation of HDL [38] and overexpression of ApoA1 may effectively inhibit the age-related decline in memory and learning ability [39]. Moreover, Kawano et al. found that the levels of both ApoA1 and ApoA2 were strikingly lower in a group of Japanese patients who suffered from late-onset nonfamilial AD [40]. Serum ApoB level had been demonstrated to be much higher in the patients with AD [41, 42], while little less is known about its function in some preclinical stages of dementia such as MCI. Kamino et al. found that there was no specific genetic association between ApoC2 and Senile dementia of Alzheimer's type [43]. The high concentration of ApoC3 in serum might lead to lipoprotein disorders and some vascular diseases such as atherosclerosis and hypertriglyceridemia; the relationship between ApoC3 and longevity is not clear yet [44, 45]. Some studies showed that the plasma level of ApoE in AD patients was much higher than the control group [46, 47]; however, others indicated that patients with AD had significantly lower level of ApoE [48, 49]. Studies about ApoJ showed that it could accelerate the progress of AD and other cognitive disorders [50]. The association between ApoH and dementia is not that clear until now.

However, most known risk factors are poor at individually discriminating risk in individuals with cognitive decline [51]. The goals of the present cross-sectional study were to examine the exact correlations of age, atherosclerosis levels, comorbidities, oxidative and inflammatory statuses, serum and plasma levels of lipid, and apolipoproteins with MMSE scores and to find out the main risk factors associated with cognitive impairment.

## 2. Material and Methods

### 2.1. Study Population

A total of 549 Han Chinese patients from Geriatrics Outpatient Clinic in Shanghai were invited to participate in a physical examination at Shanghai Huadong Hospital from June 1, 2012, to July 31, 2012. Each participant recruited was with a complete medical history. After excluding the female subjects because of the insufficient sample number (*n* = 60) and 49 older men who failed to finish the MMSE test due to either medical or psychological conditions, such as severe hearing loss, acute inflammation, and psychoneurosis, 440 male subjects (65 to 97 years old) were included in this cohort study. This study was approved by the ethics committee of Huadong Hospital of Shanghai Medical College, Fudan University, and written informed consent was obtained from all participants. Subjects in the experiment had more than 12 years of education and similar lifestyles (with no addiction of cigarettes and alcohol).

### 2.2. Assessments and Measurements

#### 2.2.1. Cognition

Cognitive function was measured by means of the MMSE, which is frequently used in screening global cognitive dysfunction and dementia [1]. The scores range from 0 to 30 and cognitive impairment is defined as a difference in score of more than one standard deviation on the MMSE (≥3 points). The subjects for the study were divided into three groups according to MMSE scores: group 1 (28 ≤ MMSE ≤ 30), group 2 (24 ≤ MMSE ≤ 27), and group 3 (MMSE ≤ 23).

### 2.3. Comorbidity Profile and Levels of Fundus Arteriosclerosis

Participants in this study were asked whether they had a physician's diagnosis of 18 chronic diseases according to the International Classification of Diseases (ICD): hypertension, dyslipidemia, obesity, diabetes, coronary heart disease, other heart diseases, venous insufficiency, stroke, epilepsy, hypothyroidism, hyperthyroidism, chronic renal disease, anemia, chronic pulmonary obstructive disease, liver disease, arthrosis, prostatic disease, and cancer. Each disease from electronic records of medical history was added to a score ranging from 0 to 18, with a higher score indicating more chronic diseases. The degree of fundus atherosclerosis (AS) was graded as levels 0, I, and II from fundus photographs according to Scheie's classification [52].

### 2.4. Serum Lipids Profiles

The serum levels of TC, TG, HDL-C, and LDL-C were determined by the standard enzymatic colorimetric technique, using Cholesterol CHOD-PAP (CHOL) and Triglyceride GPO-PAP (TG) Kits (Roche Diagnostics GmbH, Mannheim, Germany) and L-Type HDL-C and L-Type LDL-C Kits (Wako Life Sciences, Inc.), respectively.

### 2.5. Multiplex Fluorescent Immunoassay for Cytokines

Serum from 2 mL fasting peripheral blood samples was collected at 2000 ×g for 10 minutes in the morning and rapidly stored at −80°C. Serum concentrations of cytokines were assayed using Bio-Plex Human 6-Plex (IL-1*β*, IL-6, IL-4, IL-10, TNF-*α*, and RANTES) Kits and Bio-Plex Human 1-Plex (TGF-*β*) Kits (Laboratories, Hercules, California, USA). The coupled beads, serum samples, antibodies, and streptavidin-PE (each 50 *μ*L) were prepared and added prewetted to the wells of a 96-well filter plate one after another after two washing procedures. Samples were run in duplicate using Bio-Plex MAGPIX (Bio-Rad Laboratories, Inc., USA). The plate was read using Bio-Plex Manager software version 6.0. As the objective concentration of most samples was too low to read in pg/mL levels, the corresponding fluorescence intensity was used as the relative concentration. (The standard curves were presented in Supplementary Figures  1(a)–1(f), in Supplementary Material available online at http://dx.doi.org/10.1155/2015/481621; the quantitative range and the intra- and interassay CV% were shown in Supplementary Table  1).

### 2.6. Lipid Peroxidation and the Activity of Antioxidant Enzymes

The lipid peroxide (LPO) concentration in serum was determined quantitatively by using an LPO Assay Kit (Nanjing Jiancheng Bio, Jiangsu, China) according to the protocol of the manufacturer. The product absorbance of a molecule of LPO and two molecules of chromogenic agent was read at a wavelength of 586 nm with TECAN Sunrise immediately after incubation at 45°C for 60 min. The concentration of LPO (*μ*mol/L) was calculated according to the following formula:

(1) LPOμmol/L=measured  OD−blanked  ODstandard  OD−blanked  OD×standard sample10 μmol/L.

Superoxide dismutase (SOD) activity was determined using an SOD Water-Soluble Tetrazolium Salt (WST-1) Assay Kit (Nanjing Jiancheng Bio, Jiangsu, China). The corresponding SOD of the 50% inhibition ratio in the response system of 20 *μ*L of SOD solution, 20 *μ*L of serum samples, and 200 *μ*L of substrate was defined as 1 activity unit (U). The change of SOD activity was determined by measuring the absorbance at 450 nm using TECAN Sunrise.

Peroxidase (POD) activity was determined using a POD Assay Kit (Nanjing Jiancheng Bio, Jiangsu, China). The corresponding POD of catalysis of 1 *μ*g of substrate H_2_O_2_ using 1 mL of serum at 37°C for 1 min was defined as 1 activity unit (U). The change of POD activity was determined by measuring the absorbance at 420 nm using TECAN Sunrise.

Catalase (CAT) activity was assessed by using a CAT Assay Kit (Nanjing Jiancheng Bio, Jiangsu, China). Dissociation of 1 *μ*mol of substrate H_2_O_2_ using 1 mL of serum at 37°C for 1 min was defined as 1 activity unit (U). The chromogenic agent ammonium molybdate, combined with surplus substrate H_2_O_2_ in the response system, produced a yellow-colored product that was measured at 405 nm using TECAN Sunrise.

Glutathione peroxidase (GSH-px) activity was assessed by using a GSH-px Assay Kit (Nanjing Jiancheng Bio, Jiangsu, China). When 4 *μ*L of whole blood reacts with the substrate H_2_O_2_ at 37°C for 5 min after deducting the effect of nonenzymatic response, a GSH concentration decline of 1 *μ*mol/L in response system was defined as 1 activity unit (U). GSH combined with the chromogenic agent dithiobisnitrobenzoic acid produced stable yellow five-glucosinolate two-nitro benzoic acid anion that was measured at 412 nm using TECAN Sunrise.

### 2.7. Serum Apolipoproteins

The levels of different apolipoproteins were detected by Bio-Plex MAGPIX platform or ELISA. ApoA1, ApoA2, ApoB, ApoC2, and ApoC3 were assayed using Human Apolipoprotein MAGNETIC BEAD PANEL Kit (Millipore, MA, US). ApoE and ApoH were assayed using Apolipoprotein E and Apolipoprotein H Human ELISA Kits (Abcam, Cambridge, UK), respectively, and ApoJ was assayed using Human Clusterin Quantikine ELISA Kit (R&D Systems China Co. Ltd.). For ELISA, serum samples were diluted 1 : 400 into diluent. Apolipoprotein standards or samples (50 *μ*L) were added per well of human apolipoprotein in a microplate, and the assay was run according to the manufacturer's protocol. After finishing the experiments, the absorbance was immediately read on a microplate reader at a wavelength of 450 nm. The mean value of the duplicate or triplicate readings for each standard and sample was calculated, and a standard curve using 8 standard concentrations on the *x*-axis and the corresponding mean 450 nm absorbance on the *y*-axis was generated. The best-fit line was determined by regression analysis using log-log or four-parameter logistic curve fit (the representative standard curve shown in Supplementary Figure  2). The unknown sample concentration from the standard curve was multiplied by the dilution factor. The average intra- and interassay CV% were 4.6% and 7.4%, respectively (Supplementary Table  1). The minimum detectable dose of apolipoprotein was typically ~0.03 *μ*g/mL. The standard added value was 0.05–0.5 *μ*g/mL. For the Milliplex 5-plex apolipoprotein assay, similar to the Bio-Plex Human 6-Plex assay with Bio-Plex MAGPIX, the coupled beads, serum samples (5 *μ*L), and antibodies were prepared according to the manufacturer's instructions. Samples were run in duplicate using the Luminex 200 System (Luminex, Austin, USA). The objective concentration unit was ng/mL. The average intra- and interassay CV% were shown in Supplementary Table  1.

### 2.8. High-Sensitivity C-Reactive Protein Measurements

The serum high-sensitivity CRP (HsCRP) concentration was determined with an HsCRP Kit (Jun Shi Bioscientific, Shanghai, China) for study subjects using an immunonephelometric assay improved to provide greater sensitivity, which has been described in detail in the literature [38]. The World Health Organization CRP reference standard was used. The intra- and interassay CV% for this assay were ≤4.0% and ≤5.0%, respectively. The technicians were blinded to the case-control status of the samples. The normal value of Hs-CRP is <0.3 mg/L. The study subjects with Hs-CRP ≥ 10 were considered as suffering from acute infection and were excluded from the study.

### 2.9. Statistical Analysis

Body mass index (BMI) was used to show weight change. Hs-CRP levels were stratified into four degrees: (1) Hs-CRP < 0.3; (2) 0.3 ≤ Hs-CRP < 1; (3) 1 ≤ Hs-CRP < 3; (4) Hs-CRP ≥ 3. All statistical analyses were performed using SPSS Version 19.0 (SPSS Inc., Chicago, IL). Pearson Chi-Squared test was used to investigate the categorical variables including Hs-CRP, fundus atherosclerosis (AS) levels, and comorbidities. For the analyses of continuous variables, all of them were tested and, if necessary, transformed to more likely approximate the normal distribution. As a consequence, the levels of ApoC2, ApoC3, ApoE, and ApoH were log transformed, and one-way ANOVA was used for examining differences on age, ApoC2, ApoC3, ApoE, and ApoH among these groups. Kruskal-Wallis test was used to analyze the other continuous variables for they could not be transformed to normal distribution successfully under any transformations. To assess whether the factors which showed significant differences among different cognitive statuses could be associated with cognitive impairment, multivariate logistic regression analysis was performed. A *P* value <0.05 was considered statistically significant.

## 3. Results

### 3.1. Demographic Information

The total sample size of this cross-sectional study is 440.  Table 1 showed the demographic information of participants in different cognitive statuses, including age, BMI, Hs-CRP, AS levels, and comorbidities. Among three categorical variables, statistics analysis showed that age changed strikingly in three groups (*P* < 0.001) and the levels of AS are associated with cognitive status (*P* < 0.001). According to our study, participants with higher MMSE score, for example, subjects in group 1 (28 ≤ MMSE ≤ 30), shared lower levels of fundus AS when compared with those in group 2 (24 ≤ MMSE ≤ 27) (*P* = 0.004) and group 3 (MMSE ≤ 23) (*P* < 0.001) (Table 1), which probably suggested that the AS degree may be aggravated with cognitive impairment. However, there were no significant disparities in Hs-CRP and comorbidities among three cognitive statuses (Table 1).

### 3.2. Changes of Serum Biomarkers in Different Cognitive Statuses

We have examined differences of serum biomarkers among three groups including 5 markers of redox homeostasis (LPO, POD, SOD, CAT, and GSH-px), 7 markers of inflammatory status (IL-1*β*, IL-4, IL-6, IL-10, TNF-*α*, TGF-*β*, and RANTES), 4 serum lipoproteins (TC, TG, HDL-C, and LDL-C), and 8 serum apolipoproteins (ApoA1, ApoA2, ApoB, ApoC2, ApoC3, ApoE, ApoJ, and ApoH) (Table 2). The result showed significant differences of antioxidant enzyme POD (*P* = 0.026), proinflammatory cytokine IL-6 (*P* = 0.001), HDL-C (*P* = 0.002), ApoA2 (*P* = 0.001), and ApoC2 (*P* = 0.005) among three groups (Table 2). Subjects in group 3 (MMSE ≤ 23) and group 2 (24 ≤ MMSE ≤ 27) had higher levels of HDL-C, POD, and IL-6 than in group 1 (28 ≤ MMSE ≤ 30), and an obvious increase in the average age could also be observed from the higher scores of MMSE to the lower scores of MMSE, indicating that age, HDL-C, POD, IL-6, ApoA2, and ApoC2 might be closely related to the changes in cognitive status. There were no significant differences in other continuous variables under different MMSE scores according to our study (Table 2).

### 3.3. The Probable Serum Markers of Cognitive Impairment

To assess which factors could be associated with cognitive impairment, we performed multivariate logistic regression analysis with the factors above. In this analysis, age, BMI, AS levels, and comorbidities were set as control variables, and group 3 (MMSE ≤ 23) was regarded as the control group. The result presented that ApoA1 and ApoA2 might actually have impacts on cognitive impairment (Table 3). ApoA1 (28 ≤ MMSE ≤ 30: OR: 1.30, 95% CI 1.01–1.67; 24 ≤ MMSE ≤ 27: OR: 1.47, 95% CI 1.11–1.94) and ApoA2 (28 ≤ MMSE ≤ 30: OR: 0.43, 95% CI 0.18–1.02; 24 ≤ MMSE ≤ 27: OR: 0.21, 95% CI 0.08–0.54) were significantly associated with cognitive function (Table 3). Participants with a higher level of ApoA1 might have a lower risk of cognitive impairment, indicating that ApoA1 might be a protective factor in the process of cognitive impairment; on the contrary, ApoA2 might be an adverse factor of cognitive performance; that is, participants with a higher level of ApoA2 had a higher risk of undergoing cognitive impairment.

## 4. Discussion

We evaluated 32 factors which were known or unknown to be risks for cognitive impairment and found out that age, levels of fundus AS, POD, IL-6, HDL-C, ApoA2, and ApoC2 were significantly changed among different cognitive statuses, and ApoA1 and ApoA2 might be independently associated with cognitive impairment after adjusting for age, BMI, levels of fundus AS, and comorbidities. Subjects with older age, a lower level of ApoA1, and a higher level of ApoA2 might have more possibilities of undergoing cognitive impairment.

Vascular factors and related diseases were the risk factors in the development of the dementia syndrome [5, 6]. Endothelium-mediated mechanisms underlay vascular dysfunction in AD pathogenesis. Retinal microvascular changes may reflect similar pathophysiological processes in the brains of patients with AD [53]. A systemic review showed that retinal microvascular changes were significantly associated with dementia, cognitive impairment, and brain imaging abnormalities in cross-sectional studies [54]. Although the method to measure retinal microvascular changes in the present study was different, our results indicated that the severity of fundus AS was a significant risk factor of cognitive impairment. Moreover, the levels of fundus AS were the main independent factors that influenced aging [52].

Oxidative stress is strongly linked to brain aging. Although the results of clinical trials with antioxidant treatments were controversial [11, 14, 15], oxidative and antioxidant statuses were closely associated with neurodegeneration in AD patients [8, 12, 13]. In the present study, although the activity of POD was significantly different among three cognitive statuses, it did not remain of statistical significance in different cognitive statuses after adjusting for other factors (Tables 2 and 3). Numerous previous studies of antioxidant enzymes, including the activity of Cu/Zn superoxide dismutase 1, GSH, and CAT in the blood of AD or MCI patients, indicated no changes [55–58]. However, the analysis of total antioxidant status in serum samples of major studies previously revealed an overall decrease in MCI or AD, even if antioxidant enzymes were found to be unchanged [8, 59–61]. The results of assays of all antioxidant enzymes obviously reflected that the antioxidant statuses were much better than the evaluation of antioxidant enzyme activities. The antioxidant status in serum includes enzymatic and nonenzymatic antioxidants, such as soluble uric acid and ascorbic acid, lipid soluble vitamin A, vitamin E, and *α*- and *β*-carotene, dietary antioxidant nutrients, and metal proteins [8].

Chronic low-grade inflammation in older adults was closely linked to cognitive decline and dementia. A long-term study (over 20 years) indicated that repeated high or increasing IL-6 was associated with cognitive impairment; CRP alterations were inconsistent with cognition, which may involve impacts of statin medications and survival effects [62]. In our study, only a higher level of IL-6 was found in groups with lower MMSE scores, while other proinflammatory and anti-inflammatory cytokines, including systemic Hs-CRP levels, were not found to be related to cognitive status (Tables 1 and 2). The majority of previous studies demonstrated that both IL-6 and CRP were significantly elevated before the clinical onset of symptoms [16–20]. In combination with our present study, the measurements of these biomarkers might have a sensitive relationship with the asymptomatic high-risk individuals in the preclinical stage. Improving the sample size of the study is necessary to confirm the relationship between IL-6, Hs-CRP, and preclinical AD, such as memory impairment and MCI in middle-aged subjects and dementia in subjects with higher age.

Although there were contradictory results, some lipid and apolipoprotein patterns had been revealed as potential determinants of dementia and predementia syndrome [63–65]. Based on a wide range of functions, such as antioxidation, anti-inflammation, and proendothelial functions, HDL-C plays an important role in preserving cognitive function under normal and age-related and progressive neurodegenerative disorders [64]. Most studies suggested high level of cholesterol as risk factor of AD and cognitive impairment [66, 67] and later dementia [68, 69]. Moreover, high level of LDL and TC was not associated with increased risk in the individuals with one or more ApoE*ε*4 alleles [66]. However, Mielke et al. conducted an 18-year longitudinal study on 70-year-old nonsmokers and found that the increased TC level in late life was associated with the decreased dementia risk [70]. This contradictory finding might be due to the different timings in TC measurements and the clinical onset of dementia [63]. Accumulating lines of evidence suggested that serum lipid levels were bidirectionally associated with cognitive impairment, including dementia and predementia syndrome. High midlife serum TC represented a risk factor for subsequent dementia, and low serum TC and LDL-C after midlife were a risk factor for late-life cognitive impairment [28, 33]. Our cross-sectional data indicated that only HDL-C showed a statistical increase in the progress of cognitive impairment (Table 2); however, the statistical significance disappeared after adjusting for other factors (Table 3).

Regarding the apolipoproteins, a previous study demonstrated that MCI subjects demonstrated lower levels of ApoA1, ApoA2, and ApoH, higher levels of ApoE and ApoJ, and a higher ApoB/ApoA1 ratio [37]. The result in the literature on plasma or serum level of ApoE in AD was inconclusive. A study indicated that the level of ApoE was significantly higher in the MCI group [37], while several studies demonstrated no association of plasma ApoE level with AD [71, 72]. A cohort study conducted on patients with AD, patients with MCI, and healthy controls showed that the plasma level of ApoE was the lowest in those who were undergoing progress from MCI to dementia [73]. Recently, a meta-analysis revealed that the level of peripheral blood ApoE was lowered in AD patients, and ApoE might be a potential risk factor for AD [65]. Our cross-sectional data indicated that only ApoA2 and ApoC2 greatly changed in different cognitive statuses (Table 2). The result was in accordance with previous studies [40]. After adjusting for other factors, ApoA1 and ApoA2 were found to be associated with cognitive impairment (Table 3). However, there was no relationship between serum ApoE level and cognitive status. The negative result should be explained carefully because we neglect the effects of ApoE genotype on serum ApoE concentration. The ApoE genotype was related to a unique biochemical profile, such as ApoE, ApoB, and C-reactive protein levels, irrespective of AD or MCI diagnosis [74]. Some studies showed that the lower ApoE levels in individuals with AD [75] and the high plasma ApoE levels in old age with cognitive impairment [76] were not accounted for by the ApoE*ε*4 allele. Other studies demonstrated that ApoE genotype significantly influenced serum ApoE concentration [37, 73, 77]. A significant trend in reduction of the levels of ApoE from *ε*2 to *ε*4 carriers or from *ε*4 noncarriers and *ε*4 heterozygote carriers to *ε*4 homozygous individuals was observed among healthy centenarians and adults, MCI, AD, and age-matched controls [37, 46, 73, 77, 78]. Actually, no consistent association of blood ApoE levels with cognitive impairment was seen when controlling for ApoE genotype [63]. For example, ApoE levels were significantly higher in the MCI group [37] and significantly lower in patients with AD [46], and no difference in ApoE levels was seen between AD and age-related control group [73, 77, 78].

Other potential limitations of this study include the following several aspects. Firstly, the present study is the cross-sectional design of the main analyses. Without a direction of causality, the association between serum apolipoprotein patterns and cognition could not have a predictive impact. Secondly, only MMSE was used as a single measure of cognitive performance. Given that the MMSE scores of group 3 are ≤ 23, this would potentially represent mild/moderate dementia or possibly other conditions affecting memory, and group 2 have MMSE range which could be consistent with mild cognitive impairment. However, to identify these conditions would require clinical diagnosis using DSM IV for AD, Petersen's criteria for MCI, and/or other clinical tools. Therefore, the three groups used in this study were likely making it hard to interpret what variation in blood biomarker levels would mean that subjects suffered from mild cognitive impairment or dementia due to AD. Thirdly, the constitution of subjects in our study was limited to elderly male individuals only, with an average education level above high school and similar lifestyles, which might bring about biases for lacking female subjects. Only male subjects were included due to the inadequate sample size (*n* = 60) of the females, which made the results apply to men only. Thus, sex failed to be a factor in the study of relationship between cognition and biomarkers. In addition, the lack of potential confounding factors, such as social and psychological factors, a sedentary lifestyle, and frailty have been reported to increase the risk of cognitive impairment [79–82].All these might result in the inconsistency between our results and the published literature on apolipoprotein levels in different cognitive statuses. Some borderline significance occurred in our study, such as BMI and ApoB (Tables 1 and 2), possibly because of these limitations.

## 5. Conclusions

In conclusion, age, fundus AS levels, HDL-C, POD, IL-6, ApoA2, and ApoC2 showed significant disparities in different cognitive statuses. However, after adjusting for age, AS levels, BMI, and comorbidity numbers, only ApoA1 and ApoA2 were found to be possible risk factors of cognitive impairment in older men.

## Supplementary Material

The Supplementary Materials contain details for the assays done in the study, including representative standard curves for cytokines (Supplementary Figure 1) and apolipoproteins (Supplementary Figure 2), quantitative range, intra and inter-CV% for the serum biomarkers (Supplementary Table 1).

## Figures and Tables

**Table 1:** Demographic information of participants in different cognitive statuses (total sample size: *N* = 440).

		MMSE scores			Total *N*	*P*
		28 ≤ MMSE ≤ 30^#^	24 ≤ MMSE ≤ 27	MMSE ≤ 23		
Age, years, *n*		76 ± 9.5, 316	83.9 ± 8.3, 93	89.1 ± 5.0, 31	440	<0.001

BMI, *n*		24.6 (22.7–26.5), 316	24.0 (22.5–27.1), 93	22.7 (21.2–25.9), 31	440	0.096^#^

AS levels^*∗*^, *n*, %	0	23 (7.3%)	1 (1.1%)	0 (0%)	24 (5.5%)	<0.001
	I	228 (72.2%)	60 (64.5%)	14 (45.2%)	302 (68.6%)	
	II	65 (20.6%)	32 (34.4%)	17 (54.8%)	114 (25.9%)	

Hs-CRP (mg/L)	<0.3	99 (32.8%)	21 (25.3%)	10 (38.5%)	130 (31.6%)	0.128
	0.3~1	149 (49.3%)	36 (43.4%)	9 (34.6%)	194 (47.2%)	
	1~3	43 (14.2%)	19 (22.9%)	6 (23.1%)	68 (16.5%)	
	≥3	11 (3.6%)	7 (8.4%)	1 (3.8%)	19 (4.6%)	

Comorbidities^*∗*^, *n*, %	1	2 (0.6%)	2 (2.2%)	0 (0%)	4 (0.9%)	0.13
	2	5 (1.6%)	1 (1.1%)	0 (0%)	6 (1.4%)	
	3	17 (5.4%)	1 (1.1%)	2 (6.5%)	20 (4.5%)	
	4	30 (9.5%)	4 (4.3%)	2 (6.5%)	36 (8.2%)	
	5	43 (13.6%)	10 (10.8%)	3 (9.7%)	56 (12.7%)	
	6	62 (19.6%)	18 (19.4%)	8 (25.8%)	88 (20.0%)	
	7	61 (19.3%)	24 (25.8%)	8 (25.8%)	93 (21.1%)	
	8	60 (19.0%)	11 (11.8%)	7 (22.6%)	78 (17.7%)	
	9	29 (9.2%)	17 (18.3%)	1 (3.2%)	47 (10.7%)	
	10	7 (2.2%)	5 (5.4%)	0 (0%)	12 (2.7%)	

One-way ANOVA was used to test the differences of age and BMI in different groups.

Pearson Chi-Squared test was used for categorical variables.

^*∗*^Fisher's Exact Test was used for examining AS levels and comorbidities.

^#^In AS levels, compared to 24 ≤ MMSE ≤ 27, *P* = 0.004; compared to MMSE ≤ 23, *P* < 0.001.

**Table 2:** Serum probable biomarkers of lipid, apolipoprotein, and oxidative and inflammatory status in participants with different cognitive statuses.

	MMSE scores						Total *N*	*P*
	28 ≤ MMSE ≤ 30	*n*	24 ≤ MMSE ≤ 27	*n*	MMSE ≤ 23	*n*		
TC (mmol/L)	4.6 (4.0–5.2)	316	4.5 (3.9–5.1)	93	4.8 (4.1–5.5)	31	440	0.273
TG (mmol/L)	1.4 (1.0–1.9)	316	1.3 (1.0–1.8)	93	1.4 (1.1–1.8)	31	440	0.268
HDL-C (mmol/L)	0.95 (0.82–1.12)	316	0.99 (0.88–1.12)	93	1.13 (0.99–1.38)	31	440	0.002
LDL-C (mmol/L)	2.78 (2.32–3.39)	316	2.73 (2.24–3.23)	93	2.89 (1.95–3.49)	31	440	0.793
LPO (*μ*mol/L)	0.70 (0.46–0.98)	298	0.64 (0.47–0.97)	89	0.71 (0.50–0.96)	31	418	0.723
POD (U/mL)	7.81 (5.59–11.41)	304	9.04 (6.42–15.40)	89	9.57 (6.39–13.27)	31	424	0.026
SOD (U/mL)	10.44 (8.79–11.80)	275	10.84 (9.42–12.14)	84	10.73 (9.73–11.71)	29	388	0.134
CAT (U/mL)	51.82 (42.98–59.56)	297	52.25 (43.72–58.05)	84	51.92 (43.36–60.11)	30	411	0.962
GSH-px (*μ*mol/L)	70.91 (59.18–79.87)	288	70.95 (48.07–79.57)	83	67.95 (44.43–82.19)	30	401	0.840
IL-1*β* ^*∗∗*^	9.0 (8.0–10.0)	301	9.0 (8.0–10.0)	85	9.0 (8.0–9.8)	29	415	0.708
IL-4^*∗∗*^	8.8 (8.0–9.0)	302	8.0 (8.0–9.0)	89	8.0 (7.5–9.5)	31	422	0.462
IL-6^*∗∗*^	21.0 (16.0–29.0)	299	26.0 (18.0–38.5)	87	27.0 (19.0–35.0)	29	415	0.001
IL-10^*∗∗*^	21.0 (17.0–25.5)	299	21.0 (16.3–25.0)	84	20.0 (16.8–23.3)	30	413	0.961
TNF-*α* ^*∗∗*^	8.0 (7.0–9.0)	302	8.0 (7.0–9.0)	88	8.0 (7.0–9.0)	31	421	0.224
TGF-*β* ^*∗∗*^	8087.0 (601.0–15318.0)	303	9093.5 (1732.5–14060.8)	88	6455.5 (2442.2–11118.0)	31	422	0.565
RANTES^*∗∗*^	585.3 (435.5–830.9)	302	589.0 (426.5–870.8)	89	670.5 (457.5–997.0)	31	422	0.699
ApoA1 (mg/mL)	1.14 (0.97–1.28)	289	1.10 (0.97–1.27)	81	1.05 (0.68–1.37)	28	398	0.545
ApoA2 (*μ*g/mL)	292.20 (247.00–358.70)	295	262.30 (199.40–313.70)	85	271.60 (217.7–364.3)	28	408	0.001
ApoB (*μ*g/mL)	60.20 (41.20–76.70)	295	60.20 (35.88–83.05)	93	47.50 (21.21–61.80)	31	419	0.079^#^
ApoC2 (ng/mL)	4.66 ± 0.25^*∗*^	299	4.57 ± 0.27	85	4.58 ± 0.22	28	412	0.005
ApoC3 (ng/mL)	5.17 ± 0.28	298	5.12 ± 0.25	86	5.10 ± 0.32	29	413	0.147
ApoE (*μ*g/mL)	1.74 ± 0.21	305	1.74 ± 0.22	89	1.79 ± 0.18	30	424	0.421
ApoJ (*μ*g/mL)	159.4 (2.56–201.7)	305	161.79 (3.58–199.88)	89	163.40 (131.74–194.13)	30	424	0.682
ApoH (ng/mL)	5.58 ± 0.11	305	5.57 ± 0.15	89	5.62 ± 0.075	31	425	0.204

Statistics details: one-way ANOVA for ApoC2, ApoC3, ApoE, and ApoH; Kruskal-Wallis test for TC, TG, HDL-C, LDL-C, LPO, POD, SOD, CAT, GSH-px, IL-1*β*, IL-4, IL-6, IL-10, TNF-*α*, TGF-*β*, RANTES, ApoA1, ApoA2, ApoB, and ApoJ.

Data of ApoA2, ApoC3, ApoE, and ApoH are presented as mean ± SD; data of TC, TG, HDL-C, LDL-C, LPO, POD, SOD, CAT, GSH-px, IL-1*β*, IL-4, IL-6, IL-10, TNF-*α*, TGF-*β*, RANTES, ApoA1, ApoA2, ApoB, and ApoJ are presented as median (first quartile–third quartile).

^*∗*^Compared to 24 ≤ MMSE ≤ 27, *P* = 0.007.

^#^Borderline significance.

^*∗∗*^As the objective concentration was too low, the fluorescence intensity was used as the relative concentration.

**Table 3:** Logistic regression analyses for biomarkers associated with cognitive status.

	28 ≤ MMSE ≤ 30 versus *∗*			24 ≤ MMSE ≤ 27 versus *∗*		
	OR	95% CI	*P*	OR	95% CI	*P*
POD	1.01	0.93–1.10	0.744	1.04	0.95–1.13	0.385
IL-6	1.01	0.98–1.05	0.352	1.01	0.98–1.04	0.420
HDL-C	0.56	0.08–3.77	0.553	0.19	0.03–1.44	0.107
ApoA1	1.30	1.01–1.67	0.045	1.47	1.11–1.94	0.008
ApoA2	0.43	0.18–1.02	0.056	0.21	0.08–0.54	0.001
ApoC2	0.99	0.98–1.02	0.815	0.99	0.97–1.02	0.496

^*∗*^Control group: MMSE ≤ 23.

Control variables: age, BMI, AS level, and comorbidities.
