# Supplementary material for: Serum Levels of ApoA1 and ApoA2 Are Associated with Cognitive Status in Older Men
Source: Biomed Res Int. 2015 Nov 23;2015:481621. doi: 10.1155/2015/481621 (PMC4670907; doi:10.1155/2015/481621)
Supplement: Supplementary file 1 — The Supplementary Materials contain details for the assays done in the study, including representative standard curves for cytokines (Supplementary Figure 1) and apolipoproteins (Supplementary Figure 2), quantitative range, intra and inter-CV% for the serum biomarkers (Supplementary Table 1). [file 481621.f1.zip › Supplemantary Figure 1.pdf]

Supplemantary Figure 1. The standard curves of the corresponding fluorescence intensity of different cytokines to objective concentration (pg/mL)

a. IL-1β

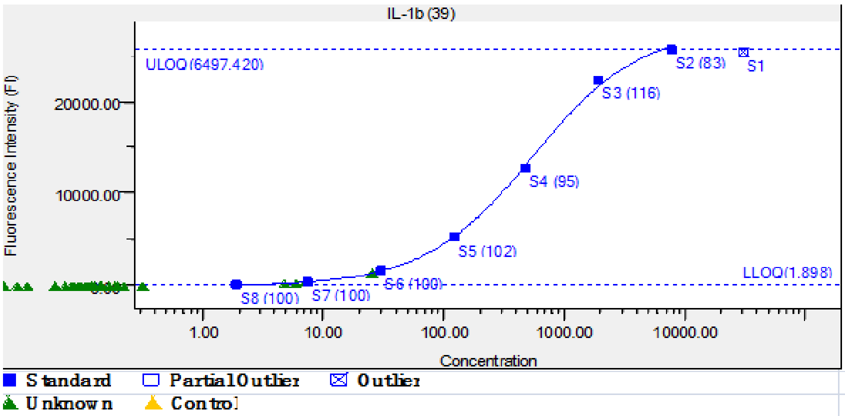

Regression Type: Logistic - 5PL
Std. Curve: FI = 0.00257759 + (27955.6 - 0.00257759) / ((1 + (Conc / 597.766)^-1.00444))^0.931699
FitProb. = 0.5856, ResVar. = 0.5352

b. IL-6

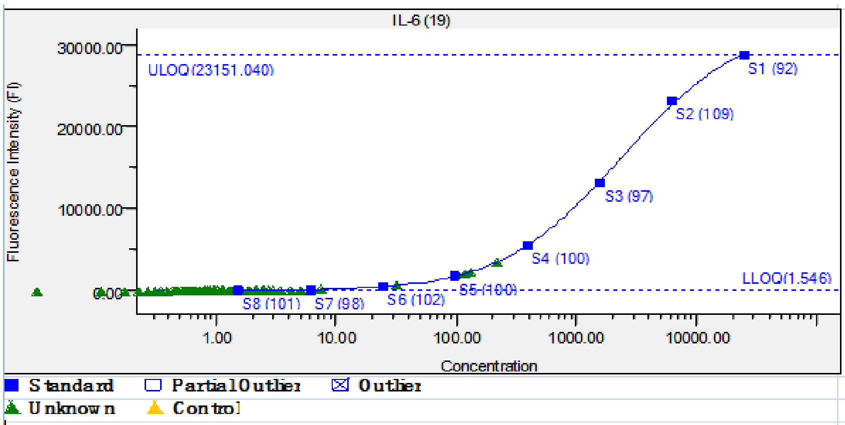

Regression Type: Logistic - 5PL
Std. Curve: FI = 7.11666 + (33472.6 - 7.11666) / ((1 + (Conc / 1871.27)^-0.803429))^1.19348
FitProb. = 0.8907, ResVar. = 0.2083

c. IL-4

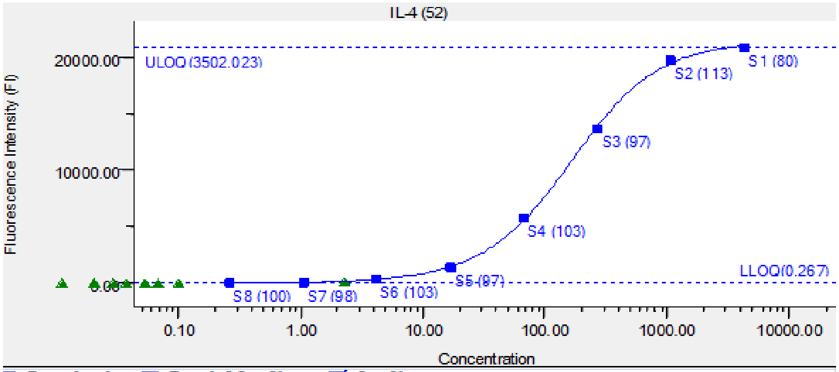

Standard Partial Outlier Outlier
Unknown Control

Regression Type: Logistic - 5PL

Std. Curve: FI = 8.94262 + (21505.1 - 8.94262) / ((1 + (Conc / 194.829)^-1.2674))^0.850943

FitProb. = 0.7829, ResVar. = 0.3587

d. IL-10

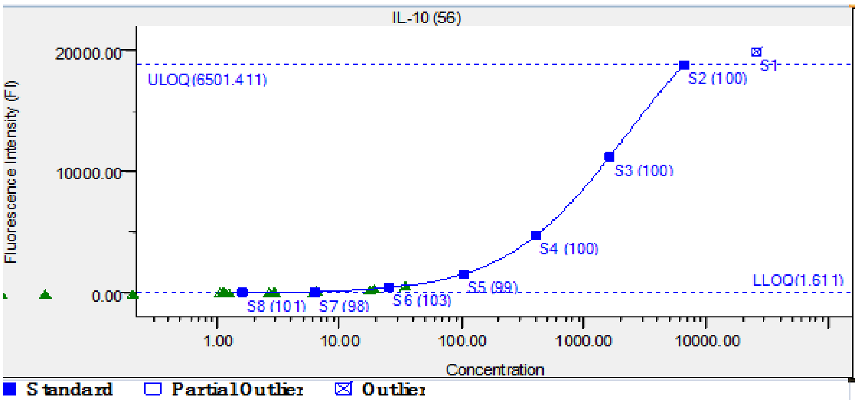

Standard Partial Outlier Outlier
Unknown Control

Regression Type: Logistic - 5PL

Std. Curve: FI = 30.5994 + (27491.1 - 30.5994) / ((1 + (Conc / 1794.73)^-0.789914))^1.21579

FitProb. = 0.8646, ResVar. = 0.1455

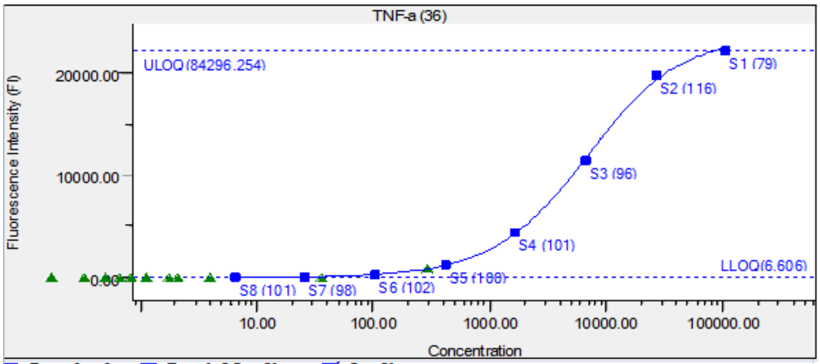

Standard Partial Outlier Outlier
Unknown Control

Regression Type: Logistic - 5PL

Std. Curve: FI = 8.46342 + (23645.3 - 8.46342) / ((1 + (Conc / 7984.71)^-1.13304))^0.876463

FitProb. = 0.8261, ResVar. = 0.2990

e. RANTES

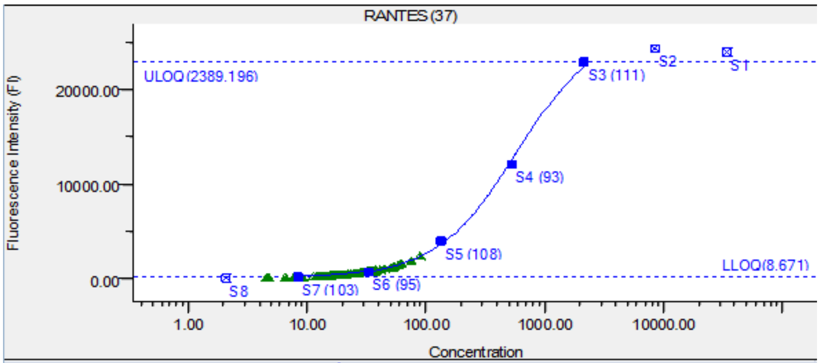

Standard Partial Outlier Outlier
Unknown Control
Regression Type: Logistic - 4PL

Std. Curve: FI = 137.692 + (26915.6 - 137.692) / (1 + (Conc / 591.699)^-1.27111)
FitProb. = 0.0249, ResVar. = 5.0336

f. TGF- β

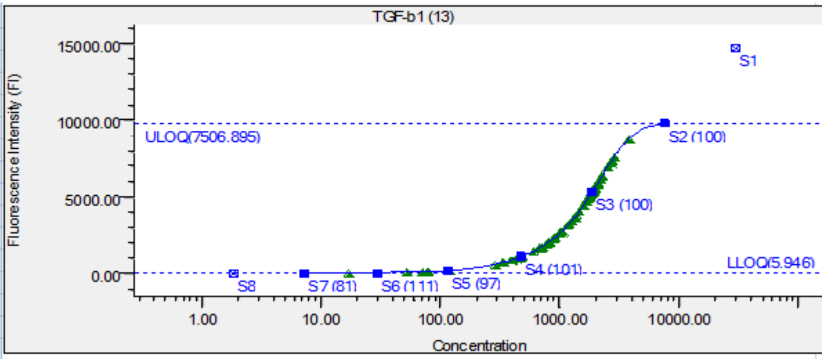

Standard Partial Outlier Outlier
Unknown Control
Regression Type: Logistic - 5PL
Std. Curve: FI = 63.0515 + (9972.96 - 63.0515) / ((1 + (Conc / 3026.63)^-3.6035))^0.330304
FitProb. = 0.3291, ResVar. = 0.9526
