# Supplementary material for: Serum Levels of ApoA1 and ApoA2 Are Associated with Cognitive Status in Older Men
Source: Biomed Res Int. 2015 Nov 23;2015:481621. doi: 10.1155/2015/481621 (PMC4670907; doi:10.1155/2015/481621)

Supplementary Fiure 2. The representative standard curve of ApoJ using 8 standard concentrations

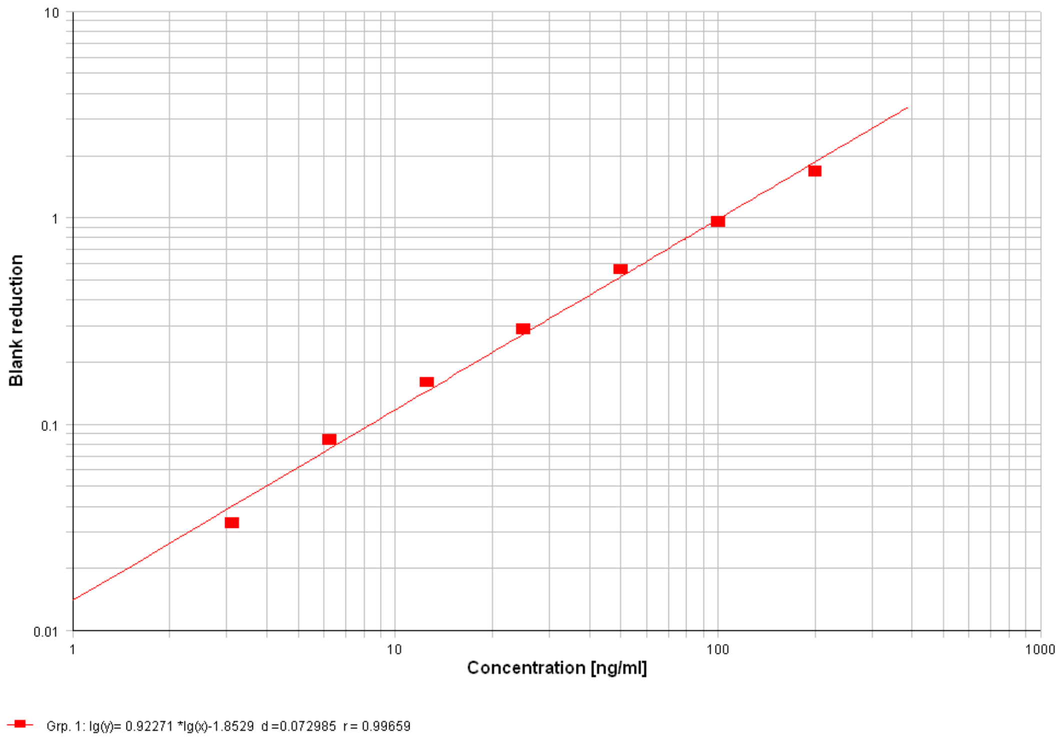

Supplement: Supplementary file 1 — The Supplementary Materials contain details for the assays done in the study, including representative standard curves for cytokines (Supplementary Figure 1) and apolipoproteins (Supplementary Figure 2), quantitative range, intra and inter-CV% for the serum biomarkers (Supplementary Table 1). [file 481621.f1.zip › Supplementary Fiure 2.pdf]
