# Supplementary material for: Serum Levels of ApoA1 and ApoA2 Are Associated with Cognitive Status in Older Men
Source: Biomed Res Int. 2015 Nov 23;2015:481621. doi: 10.1155/2015/481621 (PMC4670907; doi:10.1155/2015/481621)
Supplement: Supplementary file 1 — The Supplementary Materials contain details for the assays done in the study, including representative standard curves for cytokines (Supplementary Figure 1) and apolipoproteins (Supplementary Figure 2), quantitative range, intra and inter-CV% for the serum biomarkers (Supplementary Table 1). [file 481621.f1.zip › Supplementary Table 1.pdf]

Supplementary Table 1. Quantitative range, intra and inter-CV% for all assays

	Quantitative Range			intra-Assay CV%	inter-Assay CV%
	LLOQ	ULOQ	Sensitivity		
TC	0.08mmol/L	20.7mmol/L	0.08mmol/L	0.8	1.7
TG	0.05mmol/L	11.3mmol/L	0.05mmol/L	1.5	1.8
IL-1β	1.9	6497.4	*	6	8
IL-4	0.27	3502.02	*	9	8
IL-6	1.55	23151.04	*	7	11
IL-10	1.61	6501.41	*	5	6
TNF-α	6.61	84296.25	*	8	6
TGF-β1	5.95	7506.9	*	4.5	4.9
RANTES	8.67	2389.2	*	9	6
LPO	Unfound	Unfound	Unfound	3.3	6.99
POD	0.5U/mL	300U/mL	0.5U/mL	1.9	5.12
SOD	0.5U/mL	122.1U/mL	0.5U/mL	5.5	3.32
CAT	0.2U/mL	24.8U/mL	0.2U/mL	1.9	4.94
GSH-px	20U/mL	330U/mL	20U/mL	3.1	4.34
ApoA1	Unfound	Unfound	1.48ng/mL	< 10	< 20
ApoA2	Unfound	Unfound	1.00ng/mL	< 10	< 20
ApoB	Unfound	Unfound	4.84ng/mL	< 10	< 20
ApoC2	Unfound	Unfound	1.22ng/mL	< 10	< 20
ApoC3	Unfound	Unfound	1.71ng/mL	< 10	< 20
ApoE	0.031μg/ml	2μg/ml	0.03μg/ml	4.7	8.9
ApoJ	3.12ng/mL	200ng/mL	1.05ng/mL	3.7	7.2
ApoH	0.625ng/mL	40ng/mL	0.6ng/mL	4.8	7.2

* fluorescence intensity was used as relevant concentration.
